# Dose Administration Aid Service in Community Pharmacies: Characterization and Impact Assessment

**DOI:** 10.3390/pharmacy9040190

**Published:** 2021-11-24

**Authors:** André Vicente, Beatriz Mónico, Mónica Lourenço, Olga Lourenço

**Affiliations:** 1 FCS-UBI, Faculty of Health Sciences, University of Beira Interior, Avenida Infante D. Henrique, 6200-506 Covilhã, Portugal; a37489@fcsaude.ubi.pt; 2 Holon Pharmacies, 1100-100 Lisboa, Portugal; beatrizcmonico@gmail.com; 3 CIDTFF, Campus Universitário de Santiago, University of Aveiro, 3810-193 Aveiro, Portugal; monicalourenco@ua.pt; 4 CICS-UBI, Health Sciences Research Centre, University of Beira Interior, Avenida Infante D. Henrique, 6200-506 Covilhã, Portugal

**Keywords:** community pharmacy, dose administration aid service, impact assessment, medication adherence, safety, waste

## Abstract

Adherence to therapies is a primary determinant of treatment success. Lack of medication adherence is often associated with medical and psychosocial issues due to complications from underlying conditions and is an enormous waste of medical resources. Dose Administration Aid Service (DAAS) can be seen as part of the solution, allowing individual medicine doses to be organized according to the dosing schedule determined by the patient’s prescriber. The most recent systematic reviews admit the possibility of a positive impact of this service. In line with this background, the study reported in this paper aimed to characterize DAAS implementation in Portugal and understand the perceptions of pharmacists and owners of community pharmacies regarding the impact of DAAS, preferred methodology types, and State contribution. The study was guided by qualitative description methodology and reported using the consolidated criteria for reporting qualitative research (COREQ) checklist. Data were collected through semi-structured interviews with 18 pharmacists and/or owners of community pharmacies. Using qualitative content analysis, we identified categories that revealed that automated weekly methodology is the preferred methodology, because of its easiness of use and lower cost of preparation. However, the investment cost was felt to be too high by the participants considering the number of potential users for implementation in practice. Participants were also unanimous in recognizing that DAAS has a very positive impact in terms of safety and medication adherence, and the majority agreed that it also helped reduce medication waste. Implications of these findings for medication adherence are discussed.

## 1. Introduction

Older people, defined as those aged 65 years and older, often have multiple chronic health problems that require ongoing monitoring and medical interventions. This, and the increasing supporting evidence regarding multi-drug regimens in the management of these chronic conditions, mean that polypharmacy is often unavoidable in older people [1]. There are several definitions of polypharmacy, with the most consensual one being the simultaneous use of five or more medications [2].

Considering these complex multi-drug regimens and the decline in cognitive and physical abilities associated with aging, it comes as no surprise that medication errors often occur and may be responsible for adverse drug events (ADEs), unplanned hospitalizations, and increased morbidity, mortality, and healthcare costs [3,4,5,6,7,8]. Therefore, guaranteeing the correct, safe, and effective use of the prescribed medication is one of the greatest challenges faced by healthcare professionals, and is also recognized by the World Health Organization (WHO) as a strategy to tackle chronic health conditions effectively [9].

Medication adherence is defined by the WHO as “the degree to which the person’s behavior corresponds with the agreed recommendations from a health care provider” [9]. It is a measure of the person’s ability to accurately follow a prescribed medication regimen.

Non-adherence represents a major risk factor in chronic conditions and has become a large burden in healthcare systems. Non-adherence can be classified into intentional—when a person deliberately decides not to take their medication, or non-intentional—due to forgetfulness, lack of understanding, complexity of the regimen, or physical limitations [1,10].

The development of effective interventions to improve adherence is a challenge many researchers and health professionals have been pursuing for decades [11]. Various authors suggest that Dose Administration Aids (DAA), especially the medication reminder packaging, may represent a simple method to help tackle non-intentional non-adherence and to help patients better fulfill their treatment [12,13,14,15,16].

A Dose Administration Aid Service (DAAS) consists of repackaging solid oral medication by a healthcare provider, mostly in a community or hospital pharmacy, in order to help patients manage their polymedication [10,17]. DAA are the devices that allow medications to be organized and stored in compartments according to a patient’s dosing schedule. DAA can be grouped into three different categories: reusable multicompartment adherence aids (so-called pillboxes or manually filled dosettes); manual or automated blister packs; and sachet systems [10,18].

Reusable multicompartment adherence aids exist in various shapes and sizes. However, the most commonly used is the 7-day format, with four subcompartments for different times of the day. They can be self-administered by the patient or filled by the caregiver or pharmacy staff, which constitute major advantages, along with its reusability. Nevertheless, there are also some significant disadvantages related to hygiene, stability of the deblistered tablets, and accuracy of manually filled aids [10].

In manual blister packs, it is a pharmacist who manually distributes the medication into a securely sealed blister pack (or it is an automated system which distributes the medication automatically, in the case of automated blister packs), therefore protecting the medication until administration time. This type of DAA is widely used around the world and the corresponding service is remunerated in several countries. Their manual production is easy and affordable for every pharmacy, though they require the implementation of rigorous quality controls before dispensing to the patient. They also have a reminder function as they allow the patient to visualize the pills that need to be taken and the ones that were already taken [10].

With sachet systems, medication for a particular date and time of the day are packed in an individual sachet, labelled with the date and time, the medicine details, and the patient’s name. They are rolled up in chronological order and prepared using an automated packing technology. Community pharmacies either outsource this service to a large-scale packing facility, or have installed technology to enable onsite packing, despite the considerable investment associated with the latter [19]. This service is mainly used for institutionalized patients and when a greater number of patients is considered. In contrast to multi-drug punch cards, sachet systems do not allow for a visual control of the taken medication, and patients (especially ambulatory) need good instructions in order to follow the proper sequence of the sachets [10].

The most recent systematic reviews admit the possibility of a positive impact of a DAAS on medication adherence, drug safety, clinical outcomes, and reduction of waste. However, these studies also highlight the fact that the existing literature is still limited and susceptible to bias [20,21,22].

The aim of this study is, therefore, to characterize DAAS implementation in Portugal and understand the perceptions of pharmacists and/or owners of community pharmacies regarding the impact of DAAS, preferred methodology types, and State contribution.

## 2. Materials and Methods

### 2.1. Study Design

The study used qualitative research and semi-structured interviews to access the perceptions and experiences of pharmacists and/or owners of community pharmacies regarding DAAS (Appendix B). Creswell (2013) [23] suggests that qualitative research is preferred to quantitative research when health science researchers seek to (a) share individual stories, (b) write in a literary, flexible style, (c) understand the context or setting of issues, (d) explain mechanisms or linkages in causal theories, (e) develop theories, and (f) when traditional quantitative statistical analyses do not fit the problem at hand. In particular, the study used qualitative description, which is considered to be especially amenable to health environments research because it provides factual responses to questions about how people feel about a particular issue, what reasons they have for using particular services or features, and the factors that facilitate or hinder use [24,25,26].

### 2.2. Participants

Participants in this study were 18 pharmacists, that were either technical directors, pharmacists responsible for the service, and/or owners of community pharmacies, who were recruited through convenience sampling. In Portugal, a technical director is the pharmacist responsible for all acts (pharmaceutical services and other) performed at a specific pharmacy. Criteria for study participation were the provision of DAAS for at least six months and having at least one user of this service at the time of data collection.

**Figure 1:**
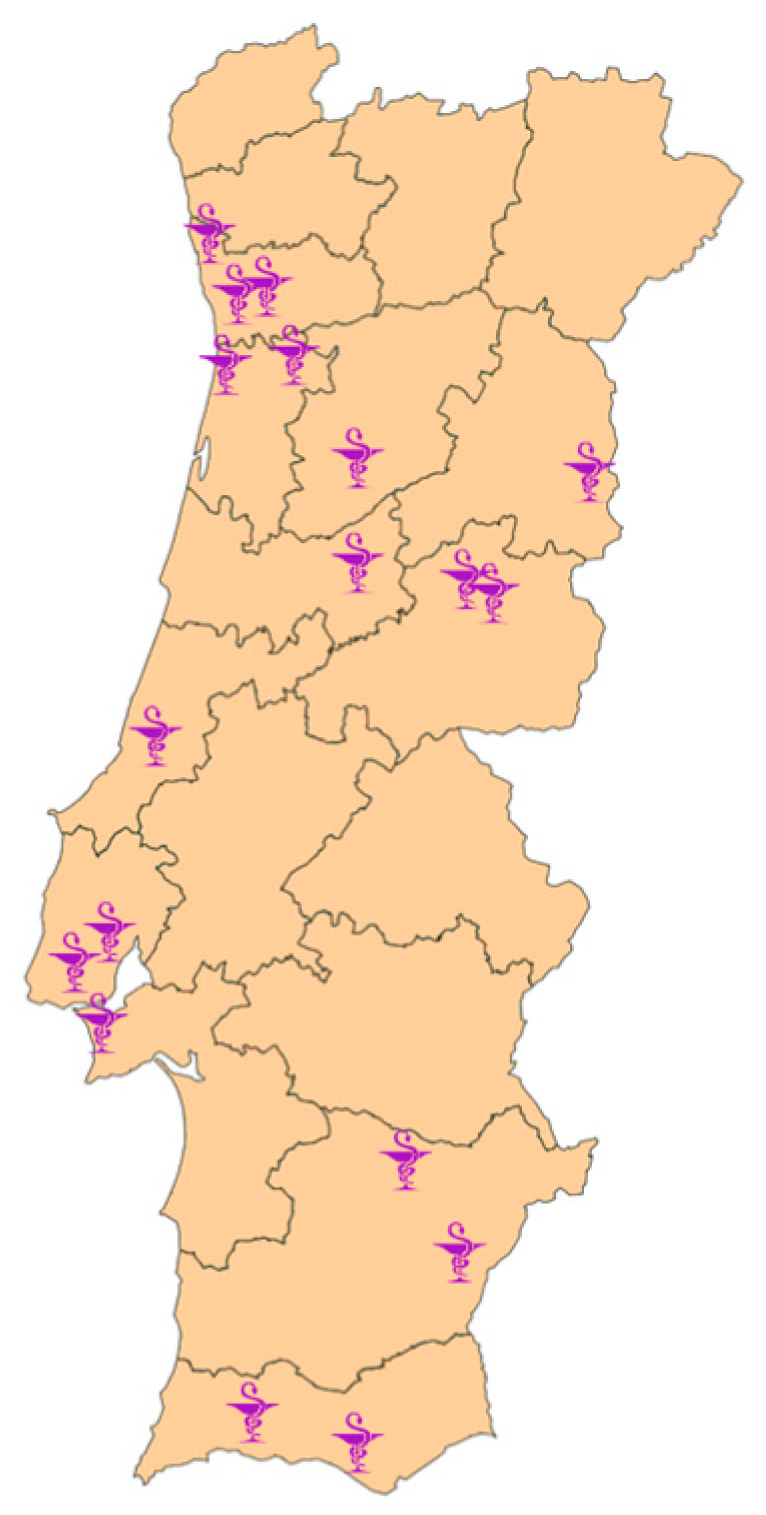
Geographic distribution of the community pharmacies.

The sample of community pharmacies was obtained in two ways: (i) through Google^®^ search, using the keywords “pharmacy” and “dose administration aid service”; (ii) via contact with pharmaceutical groups established in Portugal, which provided a list of community pharmacies that met the requirements and were interested in participating in the study. This process resulted in the identification of 433 community pharmacies, which were approached through direct contact via email and/or telephone. After being contacted twice, a reply was received from 25 pharmacies of which only 18 agreed to participate. The remaining seven pharmacies refused to participate for not having the service available at the time. The geographical distribution of the 18 pharmacies is presented in Figure 1, encompassing 11 of the 18 districts of mainland Portugal.

The pharmacists (technical directors or those responsible for the service) and/or owners of the 18 community pharmacies were interviewed by one of the researchers (AV). The participants’ profile is provided in Table 1.

All participants provided their written informed consent to participate in this study and for the publication of data included in this article. The information provided in the consent form explained the objectives of the study, the voluntary nature of participation, the possibility to withdraw from the study at any time, the materials, methods, and procedure to collect and analyze the data, and the anonymity and privacy statements. Ethical review and approval were obtained from the Ethics Committee of the University of Beira Interior (process no. CE-UBI-Pj-2021-004:ID530).

### 2.3. Data Collection

Data were collected through semi-structured, one-on-one interviews with the 18 pharmacists and/or owners of community pharmacies between 16 March 2021 and 7 July 2021. Interviews took around 40 min and were conducted via videoconference in the participants’ workspaces. All interviews were carried out by one of the researchers (AV) following a guide (Appendix A) that was developed based on the phenomenon of interest and drawn from relevant literature [27,28,29]. The interview guide included close-ended questions, which covered, for instance, the number of years the pharmacy had been providing DAAS, the number of people using the service, the methodologies available, or the average cost of the service. Open-ended questions were also used to encourage participants to express their personal opinions regarding their preferred methodology or the impact of the service on user safety, waste reduction, and therapy adherence.

The guide was tested for face and content validity by a panel of experts [30], which included 2 pharmacists familiar with the research subject. The panel assessed the appropriateness and comprehensiveness of the interview guide contents in relation to the aims and the subjects of the study. Question items were also reviewed for readability, clarity, and comprehensiveness [31]. The experts’ comments were discussed and analyzed critically by two researchers involved in this study (AV and OL) and changes for improvement were negotiated. These included reformulating some items for greater clarity, re-ordering, and adding more questions. To ensure understandability of the questions during the interviews, the participants were given the freedom to raise concerns, skip any question, or even withdraw from the interview at any time during the study without giving reasons. Furthermore, if the question was not properly comprehended, the interviewer explained it in further detail or using alternate expressions.

Considering the ethical and legal issues involved in collecting and retaining visual or audio-recorded data, only field notes were made during and immediately after the interviews. To ensure accuracy and comprehensiveness of the data, the researcher conducting the interviews (AV) made sure to use factual and objective terms, include specific quotes, and refrain from adding his own inferences and beliefs to the interview notes to minimize bias. Furthermore, caution was also taken to ensure that the notes translated participants’ perceptions or opinions by further inquiring along the views they presented. After each interview, field notes were examined and checked for accuracy, legibility, completeness, and clarity by two researchers (AV and OL).

### 2.4. Data Analysis

Data collected from the field notes were treated using content analysis, a technique commonly used in qualitative research to systematically and objectively analyze words or phrases in text documents. Hsieh and Shannon (2005) present three types of content analysis, any of which could be used in a qualitative descriptive study [32]. Conventional con-tent analysis is used in studies that aim to describe a phenomenon where existing research and theory are limited; directed content analysis is used in studies where existing theory or research exists; while summative content analysis is used to quantify and interpret words in context, exploring their usage. In this study, conventional content analysis was preferred to the other types of content analysis, considering the dearth of research on DAAS in the Portuguese context and the possibility of gaining direct information from the study participants.

**Figure 2:**
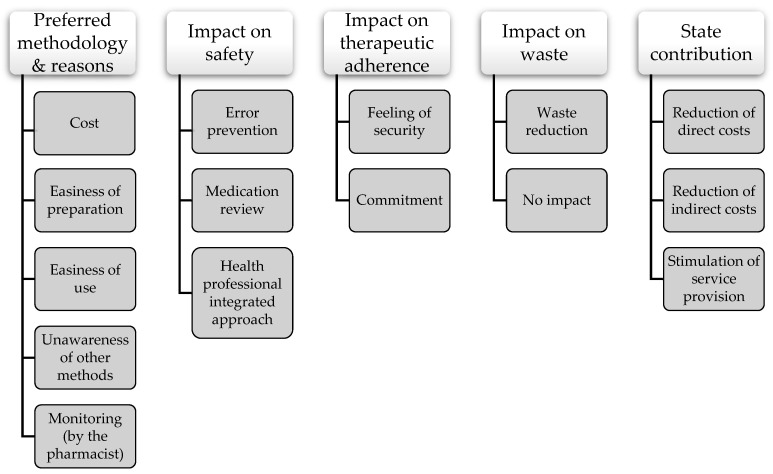
Tree diagram of categories and subcategories.

Data analysis started with reading all field notes repeatedly to gain familiarity with the content and obtain a sense of the whole. Then, data were read word by word to derive codes. Firstly, the exact words from the text that appeared to capture key thoughts or concepts related to the participants’ perceptions and experiences were highlighted. Then, notes were taken to document first impressions. As this process continued, an initial coding scheme was created by assigning labels for codes that were reflective of more than one key thought. Codes were then sorted into categories and, later, into subcategories for a more comprehensive analysis. At this stage, a tree diagram was developed to help organize these categories into a hierarchical structure (Figure 2). Exemplars for each category and subcategory were identified to facilitate reporting of the findings. To provide guidance during the reporting of this study, the consolidated criteria for reporting qualitative research (COREQ) checklist (Appendix B) was used.

It is worth mentioning that qualitative content analysis was complemented with statistical analysis, whenever appropriate. This is a common strategy in qualitative descriptive studies if they aim to more adequately or fully describe the participants or phenomenon of interest [24]. In the case of this study, descriptive quantitative analysis was used to allow for a more thorough characterization of DAAS in Portugal.

### 2.5. Trustworthiness and Reflexivity

The criteria of credibility, dependability, transferability, and confirmability were used to assess the trustworthiness of the data [33]. Credibility was ensured through periodical peer debriefing between the researchers to discuss data analysis and findings. To ensure dependability, two researchers (AV and ML) discussed the process of data analysis and codes, making appropriate adjustments as necessary to establish consensus and guarantee consistency. Furthermore, an audit trail of the research process was maintained through detailed documentation of the coding meeting notes, the recruitment protocol, and all field notes taken during and after the interviews. Transferability and rigor were achieved through data obtained from interviews with participants from 18 community pharmacies representing 11 of the 18 districts of Portugal, which allowed for a diverse range of perspectives on the phenomenon under study. To facilitate transferability, the research context, participants, and settings are described in a rich manner. Confirmability was ensured through a detailed methodological description and through reflexivity [34], meaning that the researchers were aware of their background and position and how these could influence the research process.

Our research team consisted of one male researcher (AV), a master’s student in Medi-cine (AV), and three female researchers (BM, ML, and OL), two non-practicing pharmacists (AV, OL), and a qualitative researcher (ML). Both AV and OL (an assistant professor) have interests in improving medication adherence. AV had taken a graduate study course on qualitative and quantitative research methods, ML has over 15 years of experience as a qualitative researcher, and OL has experience in conducting qualitative research. Throughout the research process, team members discussed their personal views on DAAS. All of the researchers had only a theoretical understanding of DAAS, never having advised or provided the service in a community pharmacy environment. AV conducted the interviews. After every interview, AV and OL appraised the interview, the appropriateness of the questions, and discussed the level of comfort of participants in answering the interview questions.

## 3. Results

### 3.1. Characterization of the DAAS

**Figure 3:**
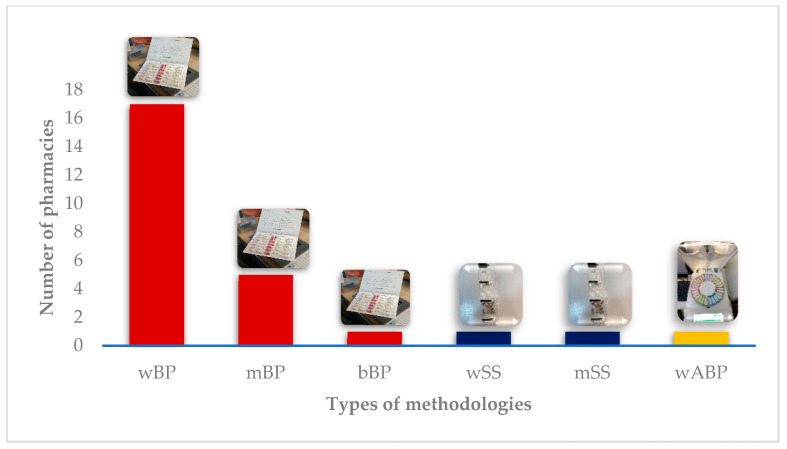
Methodologies provided. wBP: weekly blister pack; mBP: monthly blister pack; bBP: biweekly blister pack; wSS: weekly sachet system; mSS: monthly sachet system; wAMD: weekly automated blister pack.

Table 2 gives an overview of DAAS’ characteristics available in each pharmacy. All pharmacies use manual blister packs, except for one, which uses only automated blister packs (Figure 3). In two pharmacies, automatized sachet systems coexist with manual blister packs. The majority of the pharmacies (16 out of 18) provide DAAS on a weekly regimen. It is important to note that therapeutic reconciliation is a crucial part of the service in all the pharmacies, being mandatory at the beginning of the process and whenever pharmacists deem it necessary.

**Figure 4:**
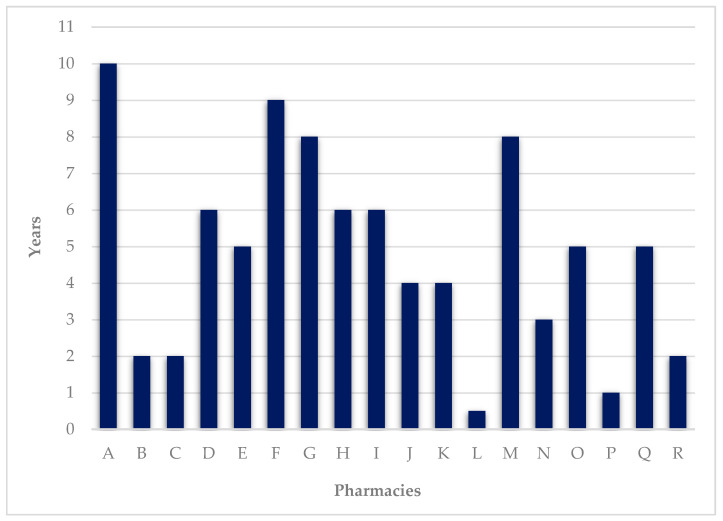
Number of years of service provision.

DAAS has been provided on average for 4.8 ± 2.77 years (median 5 years). The maximum value recorded was 10 years and the minimum was half a year (Figure 4).

**Figure 5:**
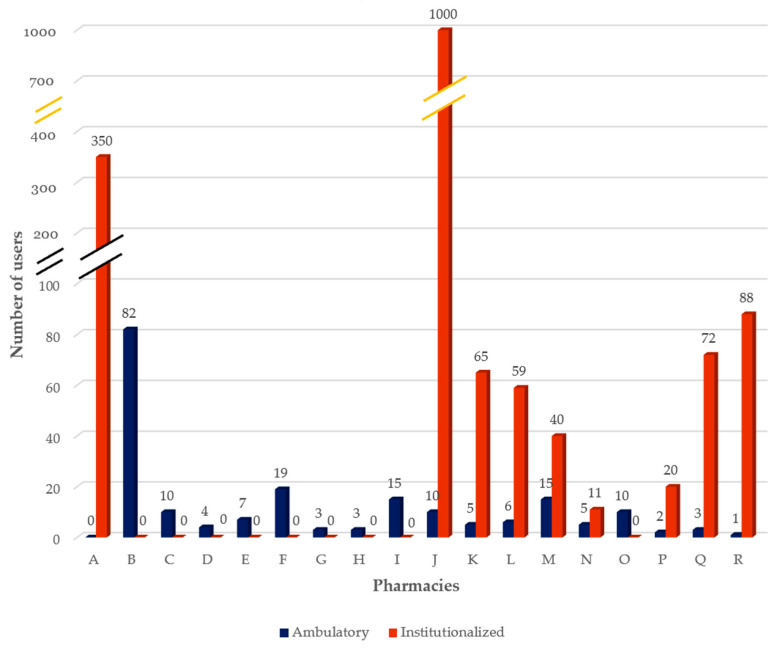
Number of DAAS current users (ambulatory and institutionalized).

At the time the interviews were made, pharmacies provided the service on average to 11 ± 18.46 ambulatory users and 95 ± 240.66 institutionalized users, showing a high variability (Figure 5). Although data suggest an apparent trend for DAAS to be a service provided mainly to institutionalized users, it is important to emphasize that only half of the pharmacies provide DAAS in this setting and, except for one, all have at least one ambulatory user. It is also worth mentioning that pharmacy C, despite not having institutionalized users at the time of the interview, has previously provided services in this type of facility.

**Figure 6:**
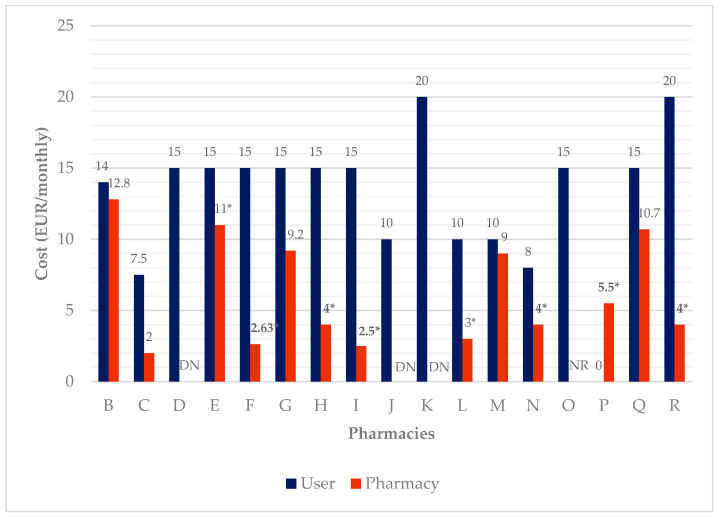
DAAS’ cost per month for users and the pharmacies. NR: did not answer; DN: do not know; * not considering human resources’ cost.

Costs for the service are summarized in Figure 6. For users, the cost is on average EUR 12.91 ± 4.89 per month. The maximum reported value was EUR 20 per month and the minimum EUR 7.5 per month. The average cost for pharmacies was EUR 8.74 per month, considering all the expenses, as reported by 5 out of 18 participants (#2, #3, #7, #13, and #17). Three participants did not know the data to properly answer this question (#4, #10, and #11), one did not want to answer (#15), and eight did not know how to quantify the human resources’ costs (#5, #6, #8, #9, #12, #14, #16, and #18). In pharmacy A, the service is provided as part of an established contract for dispensing medicines. Pharmacy P offers the service cost-free.

### 3.2. Preferred Methodology

This category captures participants’ perceptions of preferred methodologies and the reasons behind them. Preferred methodologies are those perceived as the best methodologies despite being currently applicable or not.

**Figure 7:**
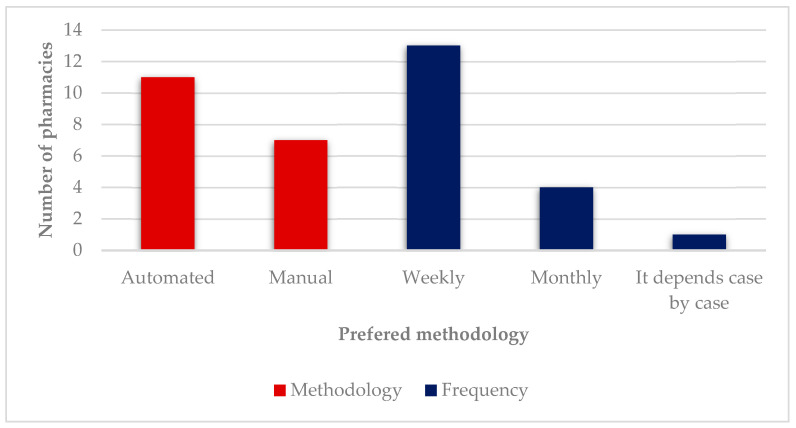
Preferred methodologies.

Although manual blister packs are the most frequently provided methodology, the majority of participants (10 out of 18) believe that an automated methodology is best in terms of convenience because of its easiness of use, and lower cost of preparation (Figure 7). Participant #17 also mentioned the easiness of the expansion of the service as an advantage. However, since “the investment costs are too high for the actual low number of users the service has, it is not practicable”. Participant #16 also added another reason for automation not being feasible, which is the “gratuitousness of the service”. Participant #18 justified the automated choice because “they did not know others”. Six participants preferred the manual methodology because of its easiness of use and one participant (#5) because “they did not know others”. In terms of frequency, a weekly methodology is preferred (13 out of 18) due to a “tighter and more rigorous monitoring by the pharmacist”. Four participants preferred monthly preparation because it was logistically easier for them. One participant (#7) did not commit with a specific answer and mentioned frequency was variable, being influenced by factors such as the “users’ cognitive ability” and “how easy it is for them to go to the pharmacy”.

### 3.3. Impact on Safety

In terms of safety, it is unanimous that DAAS improves medication safety amongst users since it “prevents errors” such as “overdose”, “forgetfulness”, “incorrect drug use”, or “wrong time or administration mode”. Participant #9 explored one of the reasons for some of these issues: “There’s a lot of confusion about generics or what is the active substance of each medication or what it’s used for”. Another participant (#10) gave the example of a patient who “stopped having neurologic complications due to medication errors after enrolling in the service”. Participants also added that “pharmaceutical monitoring is a safety net” since there is a therapeutic reconciliation process throughout the service. As participant #6 explained: “Considering that the majority of DAAS’ users are polymedicated, it is understandable that there’s an increase in terms of medication errors and side effects. So, the therapeutic reconciliation made by the pharmacist along with the therapeutic reconciliation made by different prescribers helps to achieve greater safety”. The same idea was stated by participant #10: “The initial ‘medication cleaning’ in DAAS prevents medication errors that users tend to commit”.

There are other reasons that explain the impact of DAAS on safety. For instance, as expressed by participant #9, “the monitoring of important parameters such as blood pressure for a person who is taking antihypertensives, for example, is included in DAAS”. Participant #12 alluded to the fact that “Even in terms of medications’ safety there is an improvement since storage and conservation conditions are guaranteed in a tighter way”. Participants also discussed the “better articulation between the attending physician and the pharmacist”, which can be seen as part of an integrated approach to medical care. As participant #2 explained, there is an “inverse feedback circuit from the pharmacy to the medical doctor about the actual reality of everyday users which allows to complement the care doctors provide, who very often feel they lack time for a thorough evaluation”. Participant #12 added “We discover more medication errors that patients are committing, which really helps in terms of prevention”. Participant #17 summed up the impact on safety and the advantages of the better articulation with the attending physician: “DAAS assures that the patient is not making mistakes alongside their medication (like overdose), that they do not forget to take their medication, that the patient has a qualified professional watchful to every possible medication’s interactions or side effects as well as decompensation of parameters and the arising of different symptoms related to a new health condition. This leads to better care since there’s an anticipated referral to the attending physician on a routine basis or in urgent episodes”.

### 3.4. Impact on Therapeutic Adherence

All participants were unanimous in stating that DAAS improves therapeutic adherence. One participant even mentioned that they “do not know of a method as good as DAAS to improve therapeutic adherence” (#2). Participant #6 added “I feel it has a significant impact because I can verify in my daily practice that users achieve better health results since there is a greater therapeutic adherence”. Even though there is no reliable data to compare adherence before and after starting the service, DAAS is considered to be a “very significative improvement” and one participant (#2) shared a 97% adherence rate among users. Some pharmacies save an adherence record based on the returned blister packs, which is very useful, however, “it is necessary to insist that the users bring the blister pack back so that adherence can be properly recorded. When patients bring the blister pack back we can check the therapeutic adherence which is very useful to notify caregivers or even medical doctors in some rare cases” (#4). Participant #14 helped to understand why it is often difficult to establish a link with the attending physician: “Although an effective articulation between pharmacists and doctors is crucial to monitor therapeutic adherence, it is not done regularly because the whole system is not designed to make it happen on a routine basis”.

The reasons why therapeutic adherence is improved were also advanced by participant #17, who explained that these are related to the “feeling of security the service provides” and “the commitment that is established”. Nevertheless, according to participant #3, “the system is not perfect because the user needs to know which day it is to know how to comply. However, in those cases, we make sure they do not forget by sending a notification”.

### 3.5. Impact on Waste

Considering the impact that DAAS has on waste, this is the only topic where there was no unanimity among the participants. The majority (15 out of 18) consider that in terms of medication waste, there is a reduction because the stock control is made by the pharmacy, reducing the accumulation and excessive consumption of medicines. Consequently, “patients do not buy every medicine the prescription has or, at least, in unnecessary amounts, creating pharmacies at home” (participants #10, #11, and #18). Participant #10 added “There is a handover of the medication management to the pharmacist allowing health gains and also financial savings for the State by waste reduction”. Participant #12 gave a personal example: “We have a patient who had 38 packages of medication at home when they first started the service, this was clearly a problem”. Additionally, 10 out of 18 also mention the indirect costs’ effect because the improvement in therapeutic adherence and safety leads to a decrease in health services’ costs. Participant #12 mentioned “The core of this indirect costs’ effect is simply the investment on prevention this service provides”. Nevertheless, participant #11 stated that “Although therapeutic adherence and prevention of errors diminish healthcare costs, it is difficult to quantify gains in waste, so they may go unnoticed”. Contrary to the majority’s opinion, two participants (#1 and #8) did not consider DAAS to have an impact on waste reduction. One participant (#6) did not provide a definite answer, explaining that they did not have the time to analyze the available data.

### 3.6. State Contribution

State contribution refers to all forms of money given, loaned, advanced, or reimbursed to the patients or the pharmacies in order to cover the cost of the service, and make it affordable. Regarding participants’ opinions about the State’s contribution to DAAS and the reasons behind it, all participants believe the State should contribute financially to some degree, so that patients, especially those with a handicap on therapeutic adherence or taking multiple drugs, can have access to this type of service. Participant #12 mentioned “It makes total sense, especially in those cases where there are adherence problems identified by doctors”. The reasons behind this opinion are based on the benefits DAAS has both for users (the impact assessment participants mentioned) and to the State. The State can save financial resources via the reduction of direct costs, as the service leads to the reduction of waste of reimbursed medicines, and indirect costs, because DAAS induces fewer complications of non-compliance to therapy and medication errors. Participant #6 summed this up in a very explanatory way: “The State contribution makes total sense, since the improvement on therapeutic adherence leads to better pathology control and decreases emergency visits and hospitalizations”. Furthermore, DAAS can promote an environmentally responsible disposal of waste: “It is important to note that by putting all empty packages into Valormed containers (an entity which manages medicines waste) we are also contributing to environmental sustainability, as we will be also reducing the health problems related to soil and water pollution by the chemicals from those medications” (#6). As stated by participant #17: “There are many people with medication managing difficulties who would benefit from this service and are unable to adhere for economic reasons. From a public health point of view, it would have a positive impact on the global improvement of the population’s health and quality of life due to increased adherence and monitoring of the therapy. In the long run it would translate into savings, as awareness about the correct taking of medication would avoid the reimbursement of thousands of boxes of medications that are left aside due to non-adherence”.

One participant (#2) also mentioned that with the State contribution, it would be possible to “stimulate the service provision”, including on institutionalized facilities that “sometimes cannot support that additional cost”. Furthermore, to make it work, participants said that “there is a need of more scientific data on DAAS’ advantages so that the State can see its true potential” (#2), “it should not be put at a maximum retail price as it happens on medicines because it excessively limits the market” (#4), and finally, “there needs to be a strong link between medical doctors and pharmacists” (participants #7, #10, and #14). In this topic, some ideas were suggested to increase the cooperation between pharmacists and attending physicians: “giving the pharmacists access to the full prescribed medication record of each patient and conversely giving doctors the possibility to navigate through the pharmacy records on therapeutic adherence, for example” (#2); “a practical system to notify the attending physician on side effects, interactions, and chronic therapeutic renewal alerts” (#1).

Participants #2 and #3 mentioned the need for quality assurance of DAAS: “Create uniform guidelines for quality assurance” (#2); “There must be a set of prerequisites concerning the provision of DAAS and the required competencies of the institutions that provide it” (#3). Participant #11 added: “Nowadays, local stakeholders’ contribution is being considered… given the lack of financial resources available it is difficult to have a centralized system. It is easier for city halls to implement such a system, but this might lead to inequalities between people living in different towns and cities in the same country”.

## 4. Discussion

This study aimed to characterize DAAS implementation in Portugal and understand the perceptions of technical directors, pharmacists, and/or owners of community pharmacies regarding the impact of DAAS, preferred methodology types, and State contribution.

DAAS is a recently provided service, which has been available on average for 4.8 years. It is important to stress that Portuguese legislation on DAAS was only passed in 2018 [35]. Following this publication, the Community Pharmacy College of the Ordem dos Farmacêuticos issued norms for service provision [36].The service is available for institutionalized and ambulatory users alike, with numbers served per pharmacy being highly variable.

In terms of DAAS characterization, a weekly manual blister pack is the most frequent methodology provided. The calculated user’s average cost per month was EUR 12.91, way above the literature reported cost the users are willing to pay for such a service (EUR 5 per month) [37]. This fact can be one of the reasons patients do not use this service, despite its advantages. The pharmacy’s calculated average cost was EUR 8.74 per month, reflecting an estimated profit of EUR 4.17 per month per user. However, it is important to emphasize that this data analysis is too simplistic, as estimation of pharmacy costs was only possible for some of the pharmacies. A previous study estimated the provider’s cost at EUR 6.76 per month [37], which is similar to the value obtained through the interviews. Furthermore, in Portugal, manufacturers supply medicines in blister packs, rather than bottles or tubs. In order to dispense into the devices, transfer of medication from blister packs increases the fill-times, and as a consequence, service cost.

In terms of the preferred methodology for service provision, weekly and automated were the top choices. However, high investment costs and a low number of current users make automation unprofitable, and hence, seldom available. It is interesting to note that pharmacies with a higher number of users, above 65, start providing an automated method in addition to the manual one. A weekly methodology is preferred because follow-up by the pharmacist is tighter and more rigorous.

Considering the impact on safety, the participants were unanimous in stating that DAAS has a positive impact, especially by reducing medication errors (overdose, forgetfulness, incorrect medicine, or wrong time and mode of administration), despite lack of objective data. This result is similar to unpublished data available for Portugal and abroad, namely from a randomized controlled trial in the United States [38], and the systematic review performed by Sinnemaki et al. [21].

The impact on therapeutic adherence is believed to be positive, but once again the participants mention the lack of time to analyze available data.

Regarding the impact on waste, the majority of the participants considered that there was a reduction of waste because the stock control is made by the pharmacy and prevents accumulation and abuse of medicines. There is also the indirect costs’ effect, as controlled patients tend to recur less to healthcare services. The most recent Portuguese study on medication waste dates back to 2007 [39]. At that time, the global waste identified in pharmaceutical units was 21.7% of the prescribed amount. About half (9.7%) was due to inadequacy of the size of the packaging(s) to the treatment instituted, and the other half (10.2%) to the non-adherence of patients to therapy [39].

Several systematic reviews on DAAS and DAA admit the possibility of positive effects on these three categories, and even a positive impact in clinical outcomes, although the quality of the available studies remains poor [20,21,22]. The study by Watson et al. stressed that organization devices may help unintentional medication non-adherence and could improve health outcomes [20]. This benefit was, however, not reflected by all studies.

As well as the potential benefits, DAAS may also introduce risks. Foremost, it introduces an additional step in the dispensing process when the pharmacist transfers prescribed medications into the DAA. Moreover, it is important to note that not all medicine can be packed in DAA, such as liquid or semi-solid formulations, medicines that degrade through air or light contact, and emergency medication. Even for solid medication, there is a paucity of data on the stability of chronic medications in DAA [40].

### 4.1. Limitations

This study is not without limitations. Firstly, considering the estimated population size, the sample size is low. According to data obtained from the Ordem dos Farmacêuticos (National Association of Pharmacists), around 10% of all pharmacies in Portugal provide the service (*n* = 366). Moreover, taking into account the results of a literature review study that provided a systematic analysis of qualitative health research from 2003 to 2017, it was considered that 20 interviews would be enough to reach saturation [41]. The current global pandemic was probably a factor that affected the high rate of non-response. Furthermore, study participants were sampled using the convenience sampling approach. Therefore, the study may not fully represent the wide range of participants’ perceptions.

Secondly, there is also a possibility of a positive bias concerning DAAS’ impact since all the participants worked in pharmacies where this service was available, and thus they may want to emphasize DAAS’ positive qualities and benefits.

Finally, our interview guide was not pilot-tested before the beginning of the study and participants did not have an opportunity to go through the transcripts to validate what was said during the interviews. Additionally, they did not have the opportunity to analyze the data to provide their feedback on the appropriateness of the codes in capturing their perceptions. Although the field notes were not sent to the participants, we did send the manuscript for checking before submission.

### 4.2. Implications for Research and Practice

The participants indicated multiple benefits for DAAS, related to both patients and the healthcare system.

Our findings suggest that it is important to further research DAAS’ impact on safety, adherence, and waste, as there is still a lack of reliable data and good-quality studies.

It is also crucial to explore the reasons behind the low rate of implementation of this service among community pharmacies and the reasons behind the lack of users’ adherence to this type of service.

Based on the participants’ experiences, the following aspects can be highlighted:

1. DAAS can have a positive impact on safety, adherence, and waste, especially for patients with complicated medication schedules, on multiple drugs, or with some level of cognitive impairment;
2. DAAS is useful both for ambulatory users and institutionalized users;
3. There is a need to make the service affordable for users, while not too cumbersome for pharmacies.

## Figures and Tables

**Table 1:** Participants’ characterization.

Participant	Pharmacy	Role	Sex
#1	A	Pharmacist	Female
#2	B	Owner	Male
#3	C	Technical director	Female
#4	D	Pharmacist	Female
#5	E	Pharmacist	Female
#6	F	Pharmacist	Female
#7	G	Pharmacist	Female
#8	H	Pharmacist	Female
#9	I	Pharmacist	Female
#10	J	Technical director	Female
#11	K	Technical director	Female
#12	L	Technical director	Female
#13	M	Technical director and owner	Female
#14	N	Technical director	Female
#15	O	Pharmacist	Female
#16	P	Pharmacist	Female
#17	Q	Technical director	Female
#18	R	Pharmacist	Female

**Table 2:** Overview of DAAS’ characterization.

Pharmacy	For How Long Has the Service Been Provided? (Years)	Number of Current Users		DAAS’ Cost (EUR/Monthly)		Methodology
		Institutionalized	Ambulatory	User	Pharmacy	
A	10	350	0	N/A	N/A	Weekly BP and monthly SS
B	2	0	82	14	12.8	Weekly BP
C	2	0	10	7.5	2	Weekly BP
D	6	0	4	15	DK	Monthly BP
E	5	0	7	15	11 *	Weekly BP
F	9	0	19	15	2.63 *	Weekly, biweekly, or monthly BP
G	8	0	3	15	9.2	Weekly or monthly BP
H	6	0	3	15	4 *	Weekly or monthly BP
I	6	0	15	15	2.5 *	Weekly BP
J	4	1000	10	10	DK	Weekly BP or SS
K	4	65	5	20	DK	Weekly BP
L	0.5	59	6	10	3 *	Weekly BP
M	8	40	15	10	9	Weekly BP
N	3	11	5	8	4 *	Weekly BP
O	5	0	10	15	NR	Monthly BP
P	1	20	2	0	5.5 *	Weekly BP
Q	5	72	3	15	10.7	Weekly BP
R	2	88	1	20	4 *	Weekly ABP

ABP: automated blister packs; BP: manual blister packs; DK: do not know; N/A: non-applicable; NR: did not answer; SS: sachet system; * not considering humans resources’ cost.

## Data Availability

The data that support the findings are available from the corresponding author upon reasonable request.

## Appendix A. Interview Guide

1. For how long has the Dose Administration Aid Service (DAAS) been available?
2. How many users currently use the DAAS in your pharmacy?
3. What methodology(ies) are available?
4. Which of the methodologies do you consider to be the best?
5. What is the average cost per month for users?
6. What is the average cost per user per month for the pharmacy?
7. Do you think that it makes sense to have a State contribution?
8. Is therapeutic reconciliation associated with DAAS?
9. What impact does this system have on user safety, bearing in mind that it is possible to associate therapy reconciliation?
10. What impact does DAAS have on reducing waste at this time?
11. What impact do you feel DAAS has on therapy adherence?
12. The legal framework in which this service is inserted follows an after-sales service. What do you think about making this pre-sale, that is, within a unitary distribution system?
13. With this system, would it be possible to reduce medicine waste?
14. Why did your establishment not participate in the experimental regime introduced by the government in 2010?

## Appendix B. COREQ Checklist

**Table A1:** Consolidated criteria for reporting qualitative research (COREQ) checklist.

Topic	Item No.	Guide Questions/Description	Location in Manuscript/ Reported on Page No
		Domain 1: Research team and reflexivity	
		Personal Characteristics	
Interviewer/facilitator	1	Which author/s conducted the interview or focus group? AV conducted all interviews alone	Materials and Methods/page 5
Credentials	2	What are the researcher’s credentials? AV MSc candidate BM MSc ML PhD OL PhD	Materials and Methods/page 6
Occupation	3	What was their occupation at the time of the study? AV Medicine master’s student BM Consultant pharmacist ML Researcher OL Assistant Professor	Materials and Methods/page 6
Gender	4	Was the researcher male or female? AV male BM, ML, and OL females	Materials and Methods/page 6
Experience and training	5	What experience or training did the researcher have? AV took a graduate course on quantitative and qualitative research ML has over 15 years of experience as a qualitative researcher OL has moderate level of experience with qualitative research	Materials and Methods/page 6
		Relationship with participants	
Relationship established	6	Was a relationship established prior to study commencement? No	
Participants’ knowledge of the interviewer	7	What did the participants know about the researcher? Participants were briefed on the purpose of the study Participants also reviewed the study information sheet before they gave written informed consent to be involved in the study	Materials and Methods/page 4
Interviewer characteristics	8	What characteristics were reported about the interviewer/facilitator? AV acknowledged being a Medicine master’s student with an interest in improving medication adherence	Materials and Methods/page 6
		Domain 2: Study design	
		Theoretical framework	
Methodological Orientation and Theory	9	What methodological orientation was stated to underpin the study? Qualitative descriptive methodology with qualitative content analysis	Materials and Methods/page 5
		Participant selection	
Sampling	10	How were the participants selected? Convenience	Materials and Methods/page 3
Method of approach	11	How were the participants approached? Recruitment involved email and telephone invitations sent to all community pharmacies that according to national data had the DAAS available	Materials and Methods/page 3
Sample size	12	How many participants were in the study? 18	Materials and Methods/page 3
Non-participation	13	How many people refused to participate or dropped out? Reasons? 433 pharmacies were contacted, but only 18 agreed to participate, 7 refused to participate for not having the service available at the time and the others did not respond after being contacted twice	Materials and Methods/page 3
		Setting	
Setting of data collection	14	Where was the data collected? The participants were interviewed by videoconference in their workplaces	Materials and Methods/page 4
Presence of non-participants	15	Was anyone else present besides the participant and researchers? No	
Description of sample	16	What are the important characteristics of the sample? Interviews were conducted between 16 March 2021 and 7 July 2021. Eighteen participants (17 females and 1 male) were technical directors, pharmacists responsible for the service, and/or owners of community pharmacies located in mainland Portugal from 11 different districts. In all cases, the service was available at least for six months with at least one user.	Materials and Methods/page 4
		Data collection	
Interview guide	17	Were questions, prompts, guides provided by the authors? Was it pilot-tested? Interviews were semi-structured, using a guide, which is attached as an appendix The guide was tested for face and content validity by a panel of experts	Appendix and Materials and Methods/page 4
Repeat interviews	18	Were repeat interviews carried out? If yes, how many? No	
Audio/visual recording	19	Did the researcher use audio or visual recording to collect the data? No	Materials and Methods/page 5
Field notes	20	Were field notes made during and/or after the interview or focus group? Field notes were made during and immediately after the interviews	Materials and Methods/page 5
Duration	21	What was the duration of the interviews or focus group? The semi-structured interviews took around 40 min	Materials and Methods/page 4
Data saturation	22	Was data saturation discussed? In the limitations section, we discussed that data saturation would have been reached with 20 interviews	Limitations/page 15
Transcripts returned	23	Were transcripts returned to participants for comment and/or correction? No, but the manuscript was sent to the participants before submission	Limitations/page 16
		Domain 3: analysis and findings	
		Data analysis	
Number of data coders	24	How many data coders coded the data? AV and ML discussed consistency of the codes and the coding process. AV coded all the transcripts.	Materials and Methods/page 5
Description of the coding tree	25	Did authors provide a description of the coding tree? Yes	Materials and Methods/page 5
Derivation of themes	26	Were themes identified in advance or derived from the data? Themes derived from the data	Materials and Methods/page 5
Software	27	What software, if applicable, was used to manage the data? None	
Participant checking	28	Did participants provide feedback on the findings? No	Limitations/page 16
		Reporting	
Quotations presented	29	Were participant quotations presented to illustrate the themes/findings? Was each quotation identified? Comments were supported with direct quotes from the participants who were anonymized by participant number	Results/pages 6 to 14
Data and findings consistent	30	Was there consistency between data presented and findings? Yes	Results/pages 6 to 14
Clarity of major themes	31	Were major themes clearly presented in the findings? Yes	Results/pages 6 to 14
Clarity of minor themes	32	Is there a description of diverse cases or discussion of minor themes? No	
